# Magnitude of undernutrition and associated factors among children with cardiac disease at University of Gondar hospital, Ethiopia

**DOI:** 10.1186/s40795-021-00449-9

**Published:** 2021-08-05

**Authors:** Mulat Asrade, Abdulkadir Shehibo, Zemene Tigabu

**Affiliations:** Department of Pediatrics and Child Health, University of Gondar Hospital, Gondar, Ethiopia

**Keywords:** Undernutrition, Malnutrition, Cardiac, Gondar, Ethiopia

## Abstract

**Background:**

Undernutrition and cardiac disease are interconnected in a vicious cycle. Little is known about the effect of undernutrition on cardiac disease among children in low- and middle-income countries (LMICs). This study aimed to assess magnitude of undernutrition and associated factors among children with cardiac disease at University of Gondar hospital, northwest Ethiopia.

**Method:**

This hospital-based cross-sectional study included children with cardiac disease presenting to the pediatric outpatient clinic at University of Gondar Hospital, Ethiopia. A self-administered questionnaire was administered to participating families, and medical records were reviewed. All participants who fulfill the inclusion criteria were included. Anthropometric measurements were made and the presence of malnutrition was diagnosed according to the WHO criteria. Associated factors of undernutrition analyzed by using binary logistic regression model. Variables with *p*-value ≤0.2 in bivariate analysis were fitted to the final multivariable analysis and those variables with *p*-value ≤0.05 were considered as having statistically significant association to the outcome variable. AOR and 95% confidence interval was calculated to assess the strength of association between the variables.

**Result:**

A total of 269 patients participated in the study. 177 (65.7%) were undernourished, of whom 96 (54.5%) were underweight, 70 (39.7%) were stunted, and 95 (53.9%) were wasted. Pulmonary hypertension (adjusted odds ratio [AOR] = 3.82, 95%CI 1.80–8.10), NYHA/modified Ross class III and IV heart failure (AOR = 4.64, 1.69–12.72) and cardiac chamber enlargement (AOR = 2.91, 1.45–5.66) were associated with undernutrition.

**Conclusion:**

Undernutrition is common among children with cardiac disease in northern Ethiopia. Children with pulmonary hypertension, high-grade heart failure, and cardiac chamber enlargement may warrant close follow-up for malnutrition.

## Introduction

Severe acute undernutrition affects 18.7 million children worldwide, and moderate acute undernutrition (MAM) affects an additional 32.8 million. Undernutrition remains one of the most common causes of morbidity and mortality among children throughout the world [1]. Assuming every cases of undernutrition as lack of availability of food due to poor socio-economic status is wrong and may be associated with adverse outcomes [2]. Cardiovascular diseases are one of the commonest medical conditions which are strongly associated with undernutrition [3–5]. The prevalence of malnutrition among children with cardiac disease varies according to the population studied. According to Okoromah and colleagues, a study done in Nigeria, reported a prevalence of undernutrition (90.4%) and severe undernutrition (61.2%). However, there are no published data showing the national prevalence of undernutrition among children with cardiac diseases from Africa and Ethiopia [6].

The relationship between underlying cardiac disease and undernutrition is multidirectional. Undernutrition is strongly associated with frequent hospitalization, recurrent infections, poor postoperative outcome and increased mortality. However, the cause of undernutrition in cardiac patients is multifactorial. Cardiac diseases can cause or worsen undernutrition due to several reasons. These could be due to decreased food intake, increased energy requirement and venous congestion of the bowel resulting in poor nutrient absorption [6–8].

Children with genetic syndromes/chromosomal disorders, advanced heart failure and pulmonary hypertension are more prone to develop growth failure and undernutrition [8–11]. Every child with cardiac diseases should be screened for growth failure and undernutrition to identify patients at high risk of poor outcomes who might benefit either from medical management or surgical interventions to prevent deterioration of congestive heart failure and improve prognosis [12]. Nutritional status assessment of cardiac patients is often neglected in majority hospitals in Ethiopia, despite the undernutrition contributed for increased morbidity and mortality of our cardiac patients [3]. This study aimed to identify the demographic and clinical characteristics of children with cardiac disease and undernutrition presenting to an outpatient clinic at University of Gondar Hospital (GUH), Ethiopia.

## Methods

This hospital-based cross-sectional study was conducted in the pediatric cardiology clinic at University of Gondar Hospital. University of Gondar Hospital is a resource- limited a teaching and tertiary care hospital in northern Ethiopia. It receives referrals from an area that encompasses more than 17 million people.

Patients under age 18 years presenting to the GUH cardiology clinic between April 1, to June 30, 2019 with echocardiographic evidence of anatomic defects, with or without functional deficiencies, were included in the study. Premature infants, children with a known genetic disorder, and children with other, non-CD chronic illnesses were excluded. Eligible families completed a pretested, self-administered, structured questionnaire. Two physicians reviewed the medical records of all participants. All methods were performed in accordance to declaration of Helenski. Informed consent was obtained from parents/legal guardians and this study was approved by the University of Gondar hospital internal review board (approval number – SOM/1209/2019).

Socio-demographic, anthropometric, clinical and echocardiographic data were collected in the questionnaire. One general practitioner and one pediatric resident obtained anthropometric measurements using standardized procedures. Cardiac diagnosis was made based on clinical evaluation and investigation including Doppler echocardiography. Pulmonary hypertension was defined using transthoracic echocardiography.

Nutritional status was assessed using weight-for-age (WFA), height-for-age (HFA), weight-for-height (WFH) and mid upper arm circumference (MUAC), and measurements were interpreted in accordance with World Health Organization (WHO) standards. The WHO global database on undernutrition recommends a cut-off z score of ≤ − 2 to classify low WHZ (wasting), low WAZ (underweight) and low HAZ (stunting) as moderate undernutrition, and a z score of ≤ − 3 SD to define severe undernutrition.

Data obtained from the study was entered, cleaned & verified using Epi-info 7. Then, the data were exported to SPSS version 22.0 for analysis. Descriptive summary like frequencies, proportions, graphs and cross tabs were used to present the study result. Bivariate regression was performed to identify clinical and demographic variables associated with undernutrition. Those variables found to be significantly associated with undernutrition were included in the multivariate regression model. Adjusted odds ratios and 95% confidence intervals (CIs) were calculated.

## Result

Two hundred sixty-nine children with CD were included in this study. One hundred forty-two (52.8%) patients were female and 49.8% were under age 5. The mean age was 9.6 years (range 1 month - 18 years). Fifty-two percent of patients lived in a rural area. 37.5% of fathers and 53.2% of mothers were illiterate, and 82.9% of families had more three family members (Table 1).

**Table 1:** Socio-demographic characteristics of children with cardiac disease (No = 269)

Variable	Frequency	Percent (%)
Sex		
Female	142	52.8
Male	127	47.2
Residence		
Rural	140	52
Urban	129	48
Age (in months)		
< 24	62	23
24–59	72	26.8
60–143	87	32.3
> =144	48	17.8
Maternal occupation		
Employed	30	11.2
Housewife	178	66.2
Merchant	21	7.8
Daily laborer& others	40	14.8
Maternal education		
Unable to read& write	143	53.2
able to read& write	42	15.6
Primary education	37	13.8
Secondary education	29	10.7
Tertiary education &above	18	6.7
Father education		
Uneducated	101	37.5
Able to read and write	61	22.7
Primary education	48	17.8
Secondary education	22	8.2
Tertiary education &above	37	13.8
Number of family members		
< =3	46	17.1
4–5	111	41.2
6–7	65	24.2
8 and above	47	17.5

Acquired heart disease (AHD) was more common than congenital heart disease (69.5% Vs 30.1%), and rheumatic heart disease was the most common form of acquired heart disease in the pediatric follow up clinic during the study period. Of participants with congenital heart disease, 68.2% had acyanotic disease and 31.8% had cyanotic disease. 16.4% of participants had pulmonary hypertension, 50.6% had congestive heart failure, and 90.3% had a history of hospitalization. (Table 2).

**Table 2:** Clinical profile of children with cardiac disease at University of Gondar specialized referral hospital, northwest Ethiopia, 2019 (No = 269)

Variables	Frequency	Percent (%)
Duration of symptom before Diagnosis (in months)		
< 6	192	71.4
> =6	77	28.6
Co morbid disease		
Yes	39	14.5
No	230	85.5
Frequency of follow up		
Monthly	166	61.7
Every 2 month	88	32.7
Every 3 month and above	15	5.6
Heart failure		
No	133	49.4
Mild	78	29
Moderate to severe	58	21.6
Chamber enlargement		
Yes	159	59.1
No	110	40.9
Pulmonary hypertension		
Yes	44	16.4
No	225	83.6
Type of cardiac disease		
Congenital	82	30.5
Acquired	187	69.5

**Fig. 1:**
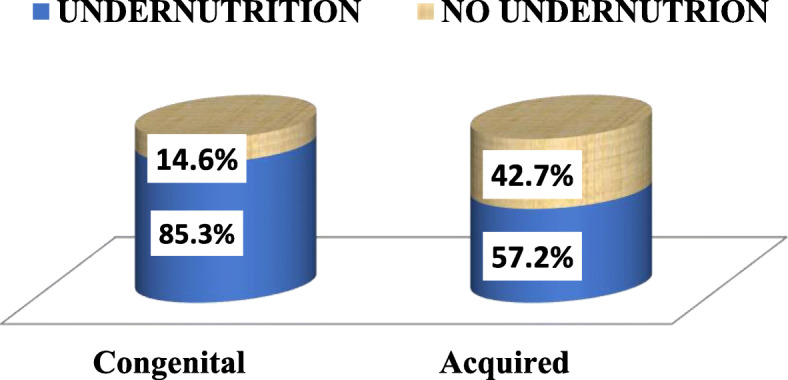
Pattern of undernutrition among children with congenital heart defect and acquired heart disease at University of Gondar specialized referral hospital, northwest Ethiopia, 2019 (No = 269)

Undernutrition was identified in 65.7% of patients, of whom 34.5% had moderate acute undernutrition and 31.2% had severe acute undernutrition. 39.7% of patients were stunted, of whom 25.2% were moderately stunted and 14.5% were severely stunted. Overall, 54.5% of patients were underweight and 53.9% were stunted. The prevalence of undernutrition was higher in children with congenital heart disease than acquired heart disease (85.7% v 57.2%, respectively) (Fig. 1).

Rural residence, recent admission, a history of > 4 admissions, congenital heart disease, and any symptoms of congestive heart failure (CHF) were associated with undernutrition on bivariate analysis. On multivariable regression, pulmonary hypertension (adjusted odds ratio [AOR] 3.79, 95% CI 1.7–12.7), moderate to severe CHF (AOR 4.6, 95% CI: 1.6–12.7), and cardiac chamber enlargement (AOR 2.91, 95% CI 1.45–5.66) were associated with undernutrition (Table 3).

**Table 3:** Multivariate logistic regression analysis of different factors associated with undernutrition among children with cardiac diseases at University of Gondar referral hospital, northwest Ethiopia, 2019(No = 269)

Variables	Undernutrition		COR with95%CI	AOR (95%CI)
	Yes	No		
Pulmonary Hypertension				
Yes	34 (77.3%)	10 (22.7%)	3.82 (1.80, 8.10)	3.79 (1.10,13.1) ^a^
No	106 (47.1%)	119 (52.9%)	1	1
Residence				
Rural	75 (58.1%)	54 (41.9%)	2.15 (1.32,3.49)	1.45 (0.74,2.86)
Urban	55 (39.3%)	85 (60.7%)	1	1
Recent admission				
Yes	59 (63.4%)	34 (36.6%)	2.54 (1.51,4.27)	1.46 (0.72,2.94)
No	71 (40.6%)	104 (59.4%)	1	1
Admission frequency				
No	10 (38.5%)	16 (61.5%)	1	1
1 to 2 times	85 (45%)	103 (55%)	1.32 (0.57,3.06)	1.06 (0.35,3.27)
Three times	14 (56%)	11 (44%)	2.04 (0.67,6.22)	1.18 (0.25,5.61)
> 4 times	20 (69%)	9 (31%)	3.56 (1.17,10.84)	1.03 (0.23,4.70)
ROSS/NYHA class				
Asymptomatic	41 (31%)	92 (69%)	1	1
Mild symptom	40 (52%)	37 (48%)	2.43 (1.36,4.33)	1.35 (0.65,2.80)
Moderate to severe	49 (84.5%)	9 (15.5%)	12.22 (5.49,27.2)	4.64 (1.69,12.72) ^a^
Cardiac chamber enlargement				
Yes	96 (61%)	62 (39%)	3.61 (2.15,6.06)	2.91 (1.45,5.66) ^a^
No	33 (30%)	77 (70%)	1	
Type of cardiac Disease				
CHD	70 (85.3%)	12 (14.6%)	1.81 (1.07,3.06)	1.47 (0.72,4.21)
Acquired heart disease	107 (57.2%)	80 (42.7%)	1	1

^a^Statistically significant; *COR* crude odds ratio, *AOR* adjusted odds ratio, *CI* confidence interval CHD, congenital heart defects; heart failure status described either by ROSS status or NYHA (New York heart association)

## Discussion

It is well known that undernutrition is common in cardiac patients and related with increased morbidity and mortality. In developing countries like Ethiopia where surgical intervention for cardiac disease like congenital heart disease and/or rheumatic heart disease is scarce or unavailable at all, the magnitude of undernutrition is expected to be high [3, 13, 14].

This study detected a high burden of undernutrition and growth failure in children with cardiac diseases. The overall prevalence of undernutrition was 65.7%, with 31.2% of cases having severe acute undernutrition. Stunting which is an indicator of chronic undernutrition was found to be 39.7% and with 14.5% of cases had severe stunting whereas the prevalence of underweight was found to be 54.5%. As noted above, this study detected a high burden of undernutrition in children with cardiac disease as compared to the country national estimate of undernutrition in Ethiopia [15]. Other studies also showed children with cardiac disease are higher risk of undernutrition compared to those without cardiac disease [5, 9]. Children with cardiac disease have several reasons to have undernutrition. This includes higher metabolic demand, inadequate intake, associated comorbidities like recurrent respiratory infection, gut dysfunction and associated chromosomal and genetic syndromes [13].

Similar to our finding, a study done in Turkey by Varun etal reported higher prevalence of acute undernutrition (65%) and chronic undernutrition (42%) [7]. Okoromah and colleagues reported a prevalence of undernutrition (90.4%), severe undernutrition (61.2%) and chronic undernutrition indicated by stunting (28.8%) in children with congenital heart disease visiting a tertiary teaching hospital Lagos, Nigeria. Though they reported lower prevalence of stunting, the overall prevalence of undernutrition and severe acute undernutrition is much higher than those seen in our study [6]. Another study in India done by Vaidyanathan and colleagues, shows higher prevalence of acute undernutrition (55.9%) evidenced by weight for height deficit [14]. This suggests the presence of heterogeneity from country-to-country accounting for the difference in determinant of undernutrition among children with cardiac disease.

This study found a higher prevalence of wasting, underweight but a lower prevalence of stunting compared to a study done at Mulago referral hospital, Uganda (31.5% wasted, 42.5% underweight and 45.4% stunted) and Cameron et al. study which reported a prevalence of acute undernutrition (33%) and chronic undernutrition (64%). In our study we included both congenital heart disease and acquired heart disease patients in contrast to the studies in the above setting where they included only children with congenital heart disease. This is could be the reason why our study has a higher prevalence of undernutrition in addition to the differences in socioeconomic characteristics and health service delivery system of these countries [16, 17].

NYHA/Modified ROSS class III and IV heart failure, cardiac chamber enlargement, and pulmonary hypertension were associated with undernutrition in our study. This is in line with various studies that reported children with advanced congestive heart failure and/or pulmonary hypertension were more likely to be malnourished [8, 9, 18]. This association may be explained by congestion of bowel and liver leading to early satiety. In addition, heart failure activates the sympathetic nervous system, leading to decreased appetite and increased caloric demand [19].

This study has several limitations. First, many patients in this study were referred to us with advanced disease, which may have led us to overestimate the true prevalence of undernutrition among children with cardiac disease. Second, we were unable to include other variables known to affect nutrition, including prematurity, genetic disorders, and previous dietary interventions. Third, our study was not powered to identify determinants of undernutrition among children with different categories of cardiac disease. However, this study provides new insights into the burden of undernutrition and its associated factors among children with cardiac disease in our hospital and encourages for a more comprehensive population-based analysis.

## Conclusion and recommendation

Undernutrition is common among children with cardiac disease in our setting. Advanced congestive heart failure, pulmonary hypertension, and cardiac chamber enlargement are associated with undernutrition. Nutritional management should be considered for all patients with cardiac disease, and prioritized for those with echocardiographic risk factors. In addition to this we recommend every effort to be made for early and definitive corrective measures to be performed including surgery.

## Data Availability

All relevant data are available within the manuscript.
